# Developments and future directions in neurodevelopmental research: A commentary on ‘camouflaging in neurodivergent and neurotypical girls at the transition to adolescence and its relationship to mental health: A participatory methods research study’

**DOI:** 10.1002/jcv2.12289

**Published:** 2024-11-27

**Authors:** Laura Crane

**Affiliations:** ^1^ Autism Centre for Education and Research (ACER) Department of Disability, Inclusion and Special Needs (DISN) School of Education University of Birmingham Birmingham UK

**Keywords:** neurodevelopmental disorders, participatory research, transdiagnostic research

As part of a broader exploration into the transition to adolescence among neurodivergent girls, McKinney et al. (in press) examined the phenomenon of camouflaging—efforts to adapt one's behaviour to appear more neurotypical—and its relationship to mental health. Collecting data from 70 neurodivergent girls with diagnoses of autism, attention deficit hyperactivity disorder (ADHD) and/or developmental coordination disorder (DCD), McKinney et al. confirmed predictions that not only is camouflaging present in this group of girls, but it was also a strong predictor of both anxiety and depression. Setting aside the importance of the findings, this research showcases two important developments in the field of neurodevelopmental research, which I will explore further in this commentary: (a) the move towards a transdiagnostic approach, and (b) the growing commitment to participatory approaches.

## MOVING TOWARDS A TRANSDIAGNOSTIC APPROACH

Traditionally, research into neurodevelopmental conditions has focussed on children and young people (CYP) grouped according to diagnostic categories (e.g., autism, ADHD, DCD). These CYP have tended to be compared with one another (in cross‐syndrome comparison studies) or have been compared against the performance of their typically developing peers. Such approaches have their roots in both clinical and academic best practice. From a clinical perspective, it was only in DSM‐5 (American Psychiatric Association [APA], 2013) that neurodevelopmental diagnoses were able to co‐occur. Prior to this change, an autism diagnosis would commonly ‘trump’ other neurodevelopmental diagnoses such as ADHD, as well as other co‐occurring diagnoses such as anxiety (see Happé & Frith, 2020, for an historical overview). From an academic perspective, neurodevelopmental research has long emphasised the importance of experimental control. For instance, in autism research, there has traditionally been a focus on recruiting autistic CYP with ‘pure’ autism, whose data would not be confounded by the presence of co‐occurring diagnoses (see Happé & Frith, 2020). Indeed, there are ongoing calls for research to move back towards the use of carefully selected samples of autistic CYP who display a more prototypical presentation of autism, advocating that such approaches may yield more consistent research findings and therefore further advance the field (Mottron, 2021).

In contrast, transdiagnostic research studies ‘focus on characteristics and mechanisms that may not align with any conventional diagnostic category’ (Astle et al., 2022, p. 398). In their review on the transdiagnostic revolution in neurodevelopmental research, Astle et al. (2022) provide several reasons why we need to move away from the longstanding focus on individual neurodevelopmental categories and towards a more inclusive, dimensional approach. These reasons include the high degree of heterogeneity *within* diagnostic categories, along with the high degree of overlap *across* diagnostic categories (Astle et al., 2022). Such observations align with the broadening of the diagnostic criteria for neurodevelopmental diagnoses, with DSM‐5 (APA, 2013) permitting ‘specifiers’ (e.g., autism *with* language delay) for the very first time. In the absence of a move towards transdiagnostic research, Fletcher‐Watson (2022, p. 418) cautions that we limit ‘the potential for real discovery, as well as practical impact on practice and in people's lives.’

Through recruiting a neurodivergent sample, comprising girls with diagnoses of autism, ADHD and/or DCD, the work of McKinney et al. (in press) is certainly aligned with this recent move towards transdiagnostic research. Further, the inclusion of girls on the waiting list for assessment was an important way of reducing the inequalities associated with access to a diagnosis (cf. Astle et al., 2022). It was notable that the girls in McKinney et al.’s neurotypical comparison group were all screened using parent‐report measures of autism, ADHD, and DCD. Interestingly, 13 participants who were originally recruited into this group, due to their parents reporting that they were neurotypical, were subsequently removed from the dataset due to meeting/exceeding cut‐off scores on the screening measures. This outcome arguably underscores the importance of using screening measures versus self‐report if researchers are categorising participants according to neurotype (i.e., neurodivergent or neurotypical), but it also raises the question of where we draw the line with respect to distinguishing neurodivergence from neurotypicality. Happé and Frith (2020) raised the controversial possibility that researchers might refrain from screening for common mental health conditions among comparison groups; akin to the move away from the quest for recruiting groups of CYP with ‘pure’ autism. As such, it was interesting to note that one of McKinney et al.’s ‘neurotypical’ participants reported a diagnosis of obsessive compulsive disorder (OCD). As it would not be pragmatic to screen for all potential indicators of neurodivergence, McKinney et al.’s approach of screening for neurodevelopmental diagnoses of focus (i.e., autism, ADHD, and DCD), while clearly documenting any other reported diagnoses, appeared to be a sensible middle ground. Such an approach also acknowledges the inherent complexity of distinguishing between neurodivergence and neurotypicality, in line with the idea that a person's needs can best be understood in terms of dimensions rather than categories (cf. Cuthbert & Insel, 2013).

McKinney et al. (in press) acknowledge that a key limitation of their research with a transdiagnostic sample was the use of a study outcome measure that was designed for one specific neurodivergent group (i.e., autistic people), to measure a construct that may be specific to autistic people (i.e., camouflaging of autistic traits). It is therefore possible that aspects of camouflaging more relevant to those with diagnoses of ADHD or DCD were not captured with the use of the adolescent version of the Camouflaging Autistic Traits Questionnaire (CAT‐Q; Hull et al., 2019), and this will be an important area to unpick in future research. Indeed, if we begin moving towards the use of transdiagnostic samples in neurodevelopmental research, a crucial next step is to work towards the development of reliable and valid outcome measures that are inclusive of a broad range of neurodivergent groups.

## A GROWING COMMITMENT TO PARTICIPATORY APPROACHES

A strength of McKinney et al.’s (in press) work was its commitment to participatory approaches. In recent years, there has been increasing awareness that research on neurodivergence does not always map onto the needs and priorities of the communities that it seeks to serve (Pellicano et al., 2014). Further, study participants have reported negative experiences of engaging in research, noting how their participation was often undervalued, their interactions with researchers were negative, and they received little, if any, feedback about the research or its outcomes (see Ashworth et al., 2021).

Participatory approaches—whereby the views of neurodivergent people and their allies shape ‘what research gets done, how it is done, and how it can be implemented’ (Fletcher‐Watson, 2022, p. 943)—have been proposed as a potential solution to this issue. However, despite reports of a ‘participatory zeitgeist’ (Palmer et al., 2019), alongside initiatives encouraging more explicit reporting of community involvement in neurodevelopmental research (e.g., Tan et al., 2024), participatory approaches are still the exception rather than the rule; particularly in relation to research whereby community members share decision‐making power equally with researchers and/or take control of the research process (Tan et al., 2024).

McKinney et al.’s (in press) research focussed on a topic that was in line with community priorities, embedded community involvement at a range of levels, and reimbursed contributors for their time and expertise. It was particularly encouraging to see such explicit reporting regarding both the methods and impact of the community involvement, since the nature and extent of reporting on community involvement is often lacking (Tan et al., 2024). As there is no one ‘correct’ way to engage in participatory research practices, McKinney et al. (in press) provide an exemplar for other researchers to reflect on, and learn from, to support their own participatory practices (cf. Pickard et al., 2022). The use of templates for participatory reporting, such as the GRIPP‐2 (Guidance for Reporting Involvement of Patients and Public; Staniszewska et al., 2017), as advocated for by Tan et al. (2024), may further serve to enhance the consistency and transparency of the reporting of participatory practices in future.

That the first author was a doctoral researcher, and that the work reported in the paper formed part of a doctoral thesis, is particularly commendable since doctoral researchers and other early career scholars often face systemic barriers to incorporating participatory practices in their work. For instance, early career researchers interviewed by Pickard et al. (2022) spoke about a lack of enthusiasm for participatory practices from the senior academics supporting them, alongside a lack of time to engage in participatory practices. It would be naïve to imply that the participatory practices reported by McKinney et al. (in press) did not require an enormous amount of time, effort, and commitment from the doctoral researcher leading the work, as well as the entire team supporting the research. Yet it is encouraging that such a project was encouraged and undertaken within the context of a doctoral degree. It is also worth acknowledging that there have been several other—excellent—participatory projects conducted by doctoral researchers in recent years. These projects (e.g., Heyworth, 2024) have yielded crucially important insights into what high quality research on neurodivergence ‘looks like’, which more established scholars could learn from. Orben (2019, p. 465) notes that: “We need all those who care about better research to stay invested, and this will not happen by telling the next generation of scientists to just sit back and hope. Early‐career researchers do not need to wait passively for coveted improvements. We can create communities and push for bottom‐up change.” McKinney et al.’s (in press) work certainly is a shining example of this push for bottom‐up change.

## CONCLUSION

Fletcher‐Watson (2022, p. 419) notes that ‘transdiagnostic research—as an ‘inherently inclusive approach—is a fertile ground for participatory methods.’ McKinney et al.’s (in press) article offers a concrete example of how future neurodevelopmental work in the field may look; with an emphasis on crossing diagnostic boundaries and embracing the complexity of neurodevelopmental diagnoses, along with a staunch focus on research that is guided by community priorities and is undertaken in collaboration with the communities it seeks to serve. By bringing these areas together, such work will hopefully lead to discoveries that better reflect, and genuinely make a difference to, neurodivergent people's day‐to‐day lives.

## AUTHOR CONTRIBUTION

**Laura Crane:** Conceptualization; Writing ‐ original draft; Writing ‐ review and editing.

## CONFLICT OF INTEREST STATEMENT

The author has declared that they have no competing or potential conflicts of interest.

## ETHICAL CONSIDERATIONS

None.

## Data Availability

Data sharing not applicable to this article as no datasets were generated or analysed.
